# *An ent*-Kaurane-Type Diterpene in *Croton antisyphiliticus* Mart.

**DOI:** 10.3390/molecules17088851

**Published:** 2012-07-25

**Authors:** Sarazete Pereira, Silvia Taleb-Contini, Juliana Coppede, Paulo Pereira, Bianca Bertoni, Suzelei França, Ana Maria Pereira

**Affiliations:** Department of Biotechnology, University of Ribeirão Preto (UNAERP), Av. Costábile Romano, 2201, CEP 14096-900, Ribeirão Preto, SP, Brazil

**Keywords:** diterpene, Euphorbiaceae, antibacterial, Cerrado

## Abstract

*Croton antisyphiliticus* is a medicinal plant widely used in the treatment of microbial infections, especially those affecting the genital tract. Crude extract, fractions and pure compound isolated from roots of this species were investigated to validate their antimicrobial activity against *Escherichia coli* and *Staphylococcus aureus*. The compound *ent*-kaur-16-en-18-oic acid was isolated as a major component (0.7% of crude extract), and its MIC value determined against *S. aureus* (ATCC 6538) was 250 μg/mL. This is the first phytochemical work on the species monitored with antimicrobial assay.

## 1. Introduction

*Croton L*. is the second largest genus of the Euphorbiaceae family, with about 1,250 species of herbs, subshrubs, shrubs, and trees [1], and approximately 350 of these species are found in Brazil [2]. Pharmacological and chemical studies of extracts and compounds isolated from *Croton* species have shown broad therapeutic action with emphasis on the antilipidemic, anti-diarrheic, immunomodulatory, antibacterial, antifungal, antiviral and anti-inflammatory activities assigned to various classes of compounds, such as volatile oil, alkaloid, proanthocyanidin, flavonoids, and mainly diterpenoid esters such as phorbols, clerodanes, labdanes, kauranes and trachylobanes [3,4,5,6,7,8].

The species C*roton antisyphiliticus* is traditionally used by the populations inhabiting the Brazilian Cerrado to treat syphilis, inflammation, genital infections and venereal cancers [9,10]. Nader *et al*. [11] reported the antimicrobial activity of extracts of some endemic plants from the Cerrado against *Staphylococcus aureus* isolated from bovine mastitic milk. So far there is no study correlating the chemical structure of *C. antisyphiliticus* compounds and antimicrobial activity. The goal of this study was to perform a phytochemical screening by evaluating the antimicrobial activity of extracts, fractions and compounds isolated from C*roton antisyphiliticus.*

## 2. Results and Discussion

**Figure 1:**
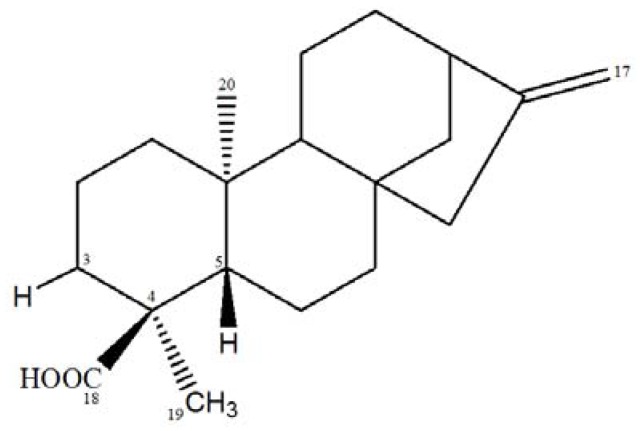
*ent*-kaur-16-en-18-oic acid.

Fractionation of the *C. antisyphiliticus* chloroform root extract resulted in the isolation of compound **1**, a white solid (0.670 mg) identified as *ent*-kaur-16-en-18-oic acid (Figure 1).

Initial identification of compound **1** was carried out on the basis of a comparison of its EIMS spectrum (70 eV) obtained by GCMS: *m/z* (rel.int. %) 302 ([M]^+^, 27) with Nist 62 mass spectral Library. The ^1^H-NMR spectrum exhibited one signal at δ 2.65 (s, H-13), two olefinic hydrogen signals at δ 4.74 (s, H-17) and 4.80 (s, H-17′), two signals of methyl groups at δ 1.06 (s, H-19) and δ 1.17 (s, H-20) and overlapping signals of methylene groups at δ 1.43–2.12. HMQC and HMBC techniques made it possible to fully attribute the kaurane skeleton to compound **1**. Thus, the ^1^H-NMR hydrogen signals at δ 4.74 (H-17) and δ 4.80 (H-17′) showed correlations in the HMQC spectrum (^1^*J*CH) with an olefinic carbon at δ 103.4, assigned to C-17. It was also observed correlations between hydrogen signals at δ 1.17 (H-20) with δ 16.5 (C-20) and δ 1.06 (H-19) with δ 18.2 (C-19) (Table 1).

The carbon of methyl group C-19 displayed long-range correlations in HMBC bidimensional spectrum with a hydrogen signal at δ 1.64 attributed to H-5. Also, the signal at δ 1.06 (s) attributed to H-19 showed correlations with carbon signals at δ 37.3 (C-3) and δ 50.6 (C-5). The signal at δ 1.71 (br s, H-3) indicated correlation with the quaternary carbon at δ 185.4, which is attributed to C-18. The ^13^C-NMR spectrum presented 20 signals, among them five quaternary (C), three tertiary (CH), ten secondary (CH_2_), and two primary (CH_3_). The chemical shift values of C-5 at δ 50.6 and C-19 at δ 18.2 made it possible to verify that carboxyl group displayed cis-equatorial position in relation to H-5 and to establish that the structure of **1** is an epimer of *ent*-kaur-16-en-19-oic acid [12]. NOESY experiments corroborate structure identification, since irradiation at δ 1.06 (H-19) produce NOE enhancements in the signal of the H-20 (δ 1.17). In the positive-ion mode, the HRESI-TOF/MS exhibited an [M+Na]^+^ ion at *m/z* 325.2140 (calculated **m/z** 325.2138), compatible with a molecular formula of C_20_H_30_O_2_. Attributions of ^1^H and ^13^C-NMR signals are summarized in Table 1. Additionally, the heteronuclear correlations between HMQC and HMBC were similar to those reported for *ent*-kaur-16-en-18-oic acid [12].

Antimicrobial activity of C. *antisyphiliticus* CHCl_3_ crude extract and fractions was insignificant compared to the antibiotic gentamicin sulphate used as positive control. Also, the pure compound *ent*-kaur-16-en-18-oic acid isolated from C. *antisyphiliticus* roots (MIC 0.250 mg/mL) was less active against *S. aureus* (ATCC 6538) than kaurenoic acid (MIC 0.125 mg/mL) (Table 2). Although kaurenoic acid has not been isolated from C*roton antisyphiliticus*, studies concerning the evaluation of pharmacological activity of chiral compounds are always relevant and recommended. 

Furthermore, the protective effect of plants against attacks by microorganisms and herbivores may be correlated with the presence of natural chiral compounds [13].

Like kaurenoic acid, *ent*-Kaur-16-en-18-oic acid is an epimer of kaurenoic acid and deviated the plane of polarized light to the left. Studies of kaurenoic acid epimers isolated from *Mikania laevigata* also showed antimicrobial activity at a level of 250 μg/mL [14]. The diterpenes *ent*-beyer-15-en-19-ol and its 4-epimer *ent*- beyer-15-en-18-ol isolated from *Helichrysum tenax* var *tenax* showed similar results in antimicrobial assays against the microorganisms tested here [15]. 

According to the literature, *ent-*kaurene compounds are inactive against Gram-negative [16] but they are active against Gram-positive bacteria [17]. Wilkens *et al*. [18] reported that this class of compounds is responsible for the lyses of protoplasts of these microorganisms.

Gil *et al*. [19] demonstrated the antimicrobial action of *ent* kaurenoic acid isolated from resin of *Pseudognaphalium vira vira* medicinal plant. According to Schmelz *et al*. [20], derivatives of *ent*-kaurene diterpenoids act as corn phytoalexins when accumulated at concentrations higher than 0.01%. Results showed that *ent*-kaur-16-en-18-oic acid represents 0.7% of the crude root extract of *C. antisyphiliticus*, meaning that this compound plays an important role in the defense against Gram-positive microorganisms present in this plant’s natural habitat, indicating its ecological importance in the protection against attacks from insects and pathogens.

## 3. Experimental 

### 3.1. General

The IR spectra were recorded on a Nicolet Protégé-460 spectrophotometer operating in a 4,000–600 cm^−1^ range in the form of KBr pellets. ^1^H- (300 and 400 MHz) and ^13^C-NMR (75 and 100 MHz) spectra were obtained on a Bruker DPX 300 or 400 in CDCl_3_, with TMS as internal reference. 2D NOESY spectra were obtained on a Bruker Avance DRX 500 MHz. The HR-ESI-MS (positive-ion mode) was recorded on a micrOTOF-Q II–ESI-TOF Mass Spectrometer Bruker Daltonics, Billerica, MA, USA. GCMS analysis was carried out using a Shimadzu (QP-2010) Gas Chromatograph Mass Spectrometer; column DB-5MS (30 m × 0.25 mm × 0.25 µm); carrier gas He; split 1:20; flow rate of 1.0 mL/min; oven program: total run time: 32 min; initial temperature at 200 °C; hold 12.00 min; ramp 10.0 °C/min to 290 °C; hold for 20.00 min; injection volume: 1 μL. Compound **1** was characterized by electron-ionization (EI) mass spectra and MS fragmentation patterns of the National Institute of Standards and Tecnology (NIST 62 lib.) spectral database. HPLC analysis was carried out by using a Shimadzu (LC-10 AD vp) diode array detector (SPD-M10A), with Supelcosil LC-18 column reversed phase (250 m × 4.6 mm, 5 μm), isocratic mobile phase of methanol/water (85:15), flow rate of 1 mL/min, and detection at 210 nm. The kaurenoic acid was provided by João Paulo Barreto de Souza from the School of Pharmaceutical Sciences, University of São Paulo.

### 3.2. Plant Material

*Croton antisyphiliticus* roots were collected in February of 2010 in Cerrado regions of Araxá, state of Minas Gerais, Brazil. A voucher specimen was deposited at the herbarium of the University of Ribeirão Preto (HPM-482). Roots were dried at 50 °C and powdered.

### 3.3. Extraction and Isolation

Powdered material (745 g) was extracted in CHCl_3_ (2 L) at room temperature for 7 days. The CHCl_3_ extract was concentrated to a small volume at reduced pressure to yield 8.93 g of oily residue. This residue was fractionated on a silica gel column (Silicagel Acrós®, 0.060–0.200mm, 60 A; Geel, Belgium), eluting with hexane, hexane/ethyl acetate, ethyl acetate and methanol gradient elution to 100%. 

The resulting fractions were analyzed by using thin layer chromatography (TLC; Aldrich®, silica gel 60 GF254 20 cm × 20 cm × 0.25 mm) and pooled according to their chromatographic profiles. This resulted in 11 fractions which were evaluated for antimicrobial activity. Fraction 4 (Fr. 4) was re-chromatographed in silica gel flash (Silicagel 60 Fluka®, 0.04–0.063 mm, 230–400 mesh; Buchs, Switzerland) by using an isocratic system of hexane/ethyl acetate (9:1) to yield 10 sub-fractions. The active sub-fraction (Fr. 4.7) was purified by HPLC, resulting in compound **1** which was identified by spectroscopic methods.

### 3.4. Determination of Antimicrobial Activity by Bio-Autographic Assay and Minimum Inhibitory Concentration (MIC)

The C*roton antisyphiliticus* crude extract CHCl_3_ and its sub-fractions were subjected to bio-autographic assay. The samples (2.0 mg) were diluted in 1 mL chloroform and applied on glass plates with silica gel F254. The plates were developed in mobile hexane/ethyl acetate phase (8:2) and then covered with Agar BHI containing 3 × 10^6^ cfu/mL of each microorganism in suspension separately. This procedure was duplicated. After 24 h at 35 °C, each bio-autogram was stained with 2,3,5-triphenyltetrazolium chloride (TTC) aqueous solution in order to observe inhibition areas. To determine the chemical profile of the samples, replication of chromatographic plate was performed simultaneously and revealed with vanillin/sulphuric acid color reagent, followed by heating.

The bio-autography confirmed the antimicrobial activity of crude extract and fractions. MIC values of crude root extracts and fractions were determined against the bacterial strains according to the CLSI M27-A2 (2003) guidelines [21] Stock cultures were submitted to serial dilutions ranging from 15.6–1000 µg.

### 3.5. Microbial Strains

The antimicrobial assay was carried out against four selected microorganisms: *Escherichia coli* ATCC 25922 and *Staphylococcus aureus* ATCC 6538 purchased from the American Type Culture Collection (Manassas, VA, USA) and two clinical isolates, *Escherichia coli* and *Staphylococcus aureus*, obtained from urinary tract and oropharynx, respectively. The final concentration of DMSO in each medium was 1.25%, which did not affect the growth of the test strains. The microorganisms were re-activated before the assay in Müller Hinton medium, cultured with no agitation at 37 °C for 1 day. The antimicrobial assay was performed by using the serial dilution method according to the CLSI M7-A8 (2009) [22]. The lowest concentration of the tested compounds in which no growth was observed was defined as MIC. The assays were performed in triplicate.

## 4. Conclusions

The isolation of *ent-*kaur-16-en-18-oic acid and validation of the antibacterial activity of this compound against *Staphylococcus aureus* corroborates other ethnopharmacological studies. 

## Figures and Tables

**Table 1:** HMQC and HMBC spectra data of compound **1** (CDCl_3_).

C/H	δC	δH	^2^*J*CH	^3^*J*CH
**C**				
4	47.9	-	H3; H19	H2; H6
8	44.7	-	H7; H9; H14; H15	H6; H11
10	39.0	-	H1; H5; H9; H20	H2; H6
16	156.1	-	H15; H17	H12
18	185.4	-	-	H5; H19
**CH**				
5	50.6	1.64 *^a^*	H6	H7; H19; H20
9	56.4	1.17 (br s)	H11	H5; H7; H12; H14; H15; H20
13	44.3	2.65(s)	H12; H14	H15; H17
**CH_2_**				
1	40.2	1.10 *^a^*; 1.95 *^a^*	H2	H3; H5; H9; H20
2	18.3	1.54 *^a^*	H1	-
3	37.3	1.54 *^a^*; 1.71 *^a^*	H2	H1; H5; H19
6	23.6	1.16 (br s); 1.48 *^a^*	H5; H7	-
7	41.0	1.44 *^a^*; 1.59 *^a^*	H6	H5; H9; H14
11	18.1	1.61 *^a^*	H9; H12	-
12	33.6	1.46 *^a^*; 1.59 *^a^*	H11	H9; H14
14	39.9	0.86 *^a^*; 1.82 *^a^*	-	H7; H9; H12; H15
15	49.4	2.05 *^a^*	-	H7; H9: H14; H17
17	103.4	4.74 (s), 4.80 (s)	-	-
**CH_3_**				
19	18.2	1.06 (s)	-	H5
20	16.5	1.17 (br s)	-	H1; H5; H9

*^a^* Overlapping signals.

**Table 2:** Antimicrobial activity of extract, fractions and diterpene of *C. antisyphiliticus*.

**Samples**	***Escherichia coli* ATCC 25999** (mg/mL)	***Escherichia coli* from clinical isolates** (mg/mL)	***Staphylococcus aureus* ATCC6538** (mg/mL)	***Staphylococcus aureus*** **from clinical isolates** (mg/mL)
Crude extract CHCl_3_	2	2	2.5	2
C1	2	2	2	2
C2	2	2	2	2
C3	2	2	2	2
C4	2	2	1	2
C5	2	2	2	2
C6	2	2	2	2
C7	2	2	2	2
C8	2	2	2	2
C9	2	2	2	2
C10	2	2	2	2
C11	2	2	2	2
*ent*-kaur-16-en-18-oic acid *	2	2	0.250	2
kaurenoic acid	1	2	0.125	2
Gentamicin sulphate **	0.007	0.125	0.007	0.015

* Purified from C*roton antisyphiliticus*; ∗∗ Purchased from Sigma Chemical Co.

## Supplementary Materials

Supplementary materials can be accessed at: http://www.mdpi.com/1420-3049/17/8/8851/s1.
