# Supplementary material for: An ent-Kaurane-Type Diterpene in Croton antisyphiliticus Mart
Source: Molecules. 2012 Jul 25;17(8):8851–8. doi: 10.3390/molecules17088851 (PMC6268975; doi:10.3390/molecules17088851)

Support Information

Figure 1. GC chromatogram of compound **1**.

Sample Information

Analyzed by	: Admin
Analyzed	: 29/5/2012 02:04:34
Sample Type	: Unknown
Level #	: 1
IS Amount	: [1]=1.000
Sample Amount	: 1.000
Dilution Factor	: 1.000
Vial #	: 18
Injection Volume	: 1.000
Data File	: C:\Amostras GCMS\Silvia Taleb\Croton NO.qgd
Method File	: C:\Amostras GCMS\Silvia Taleb\Triterpeno DB5.qgm
Org Method File	: C:\Amostras GCMS\Silvia Taleb\Triterpeno DB5.qgm
Report File	:
Tuning File	: C:\GCMSsolution\System\Tune1\24-05-2012.qgt
Modified by	: Admin
Modified	: 29/5/2012 08:44:24

Chromatogram C:\Amostras GCMS\Silvia Taleb\Croton NO.qgd

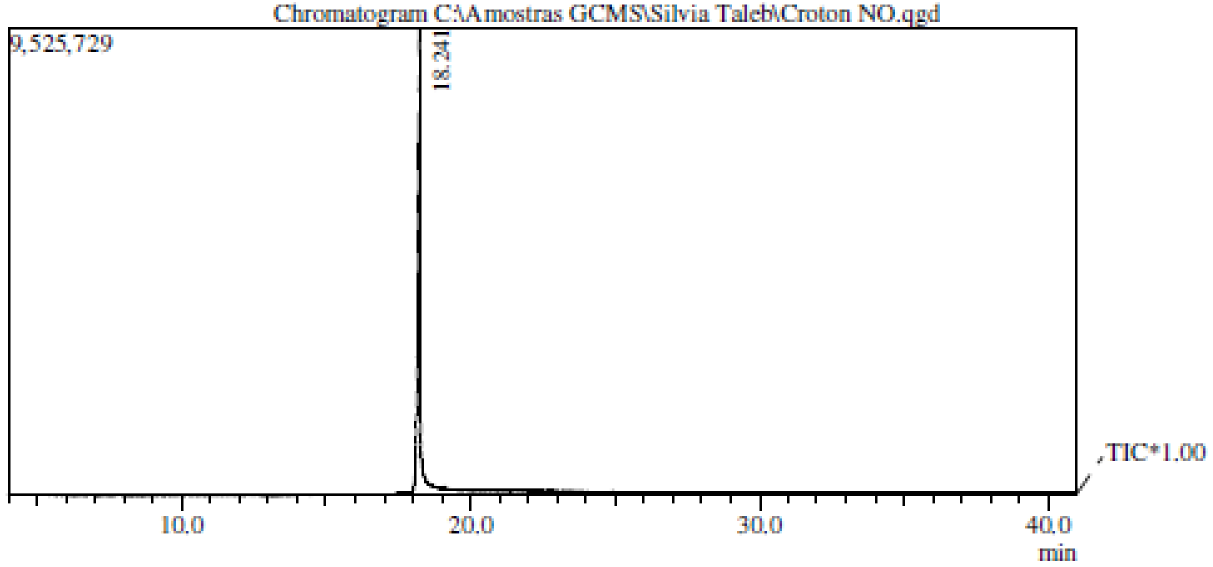

Peak Report TIC

Peak#	R.Time	Area	Area%	Name	Base m/z
1	18.241	50730346	100.00		259.15
		50730346	100.00		

Figure 2. EI-MS spectrum of compound **1**.

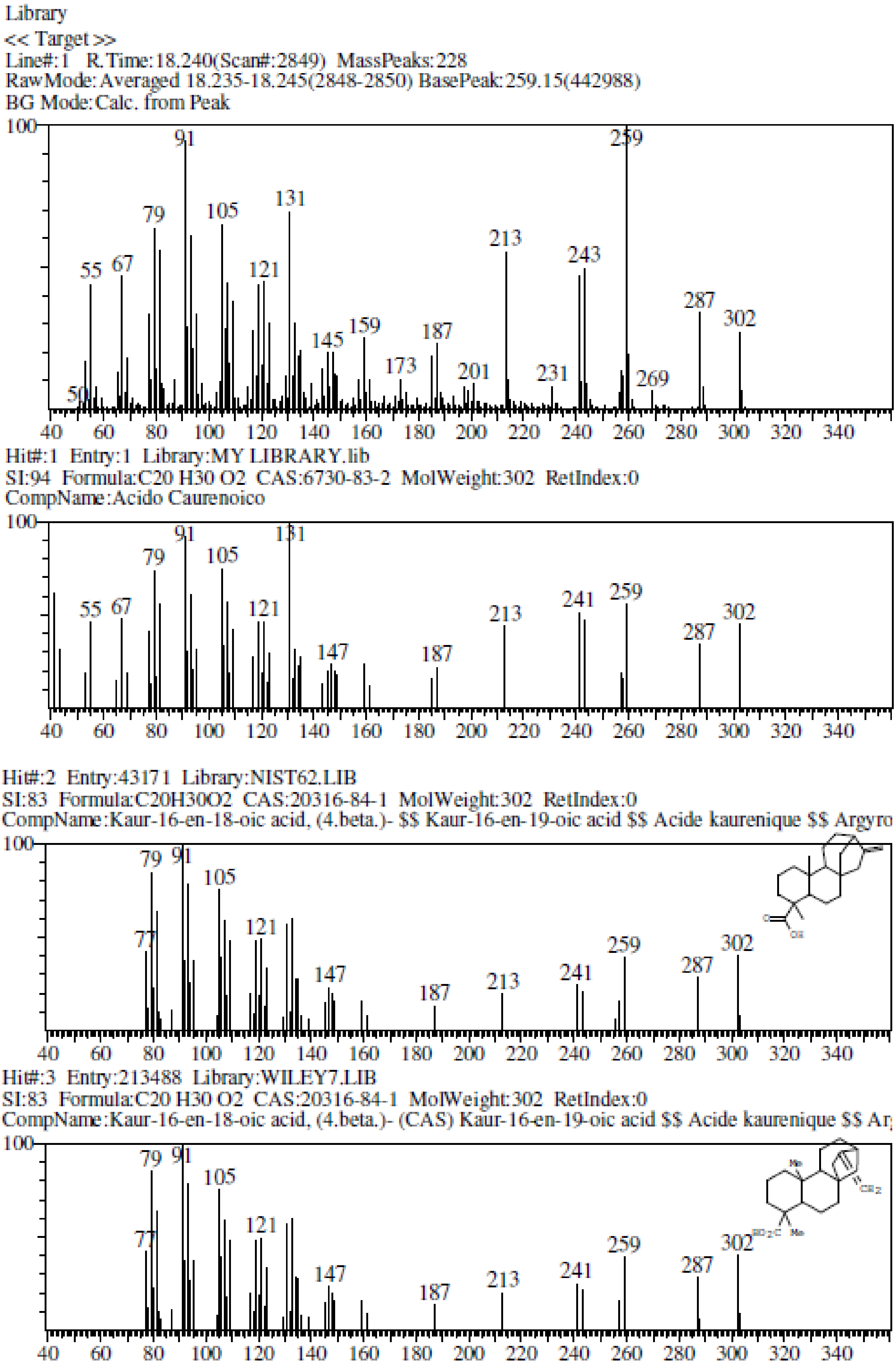

Figure 3. HR-ESI-MS spectrum of compound **1**.

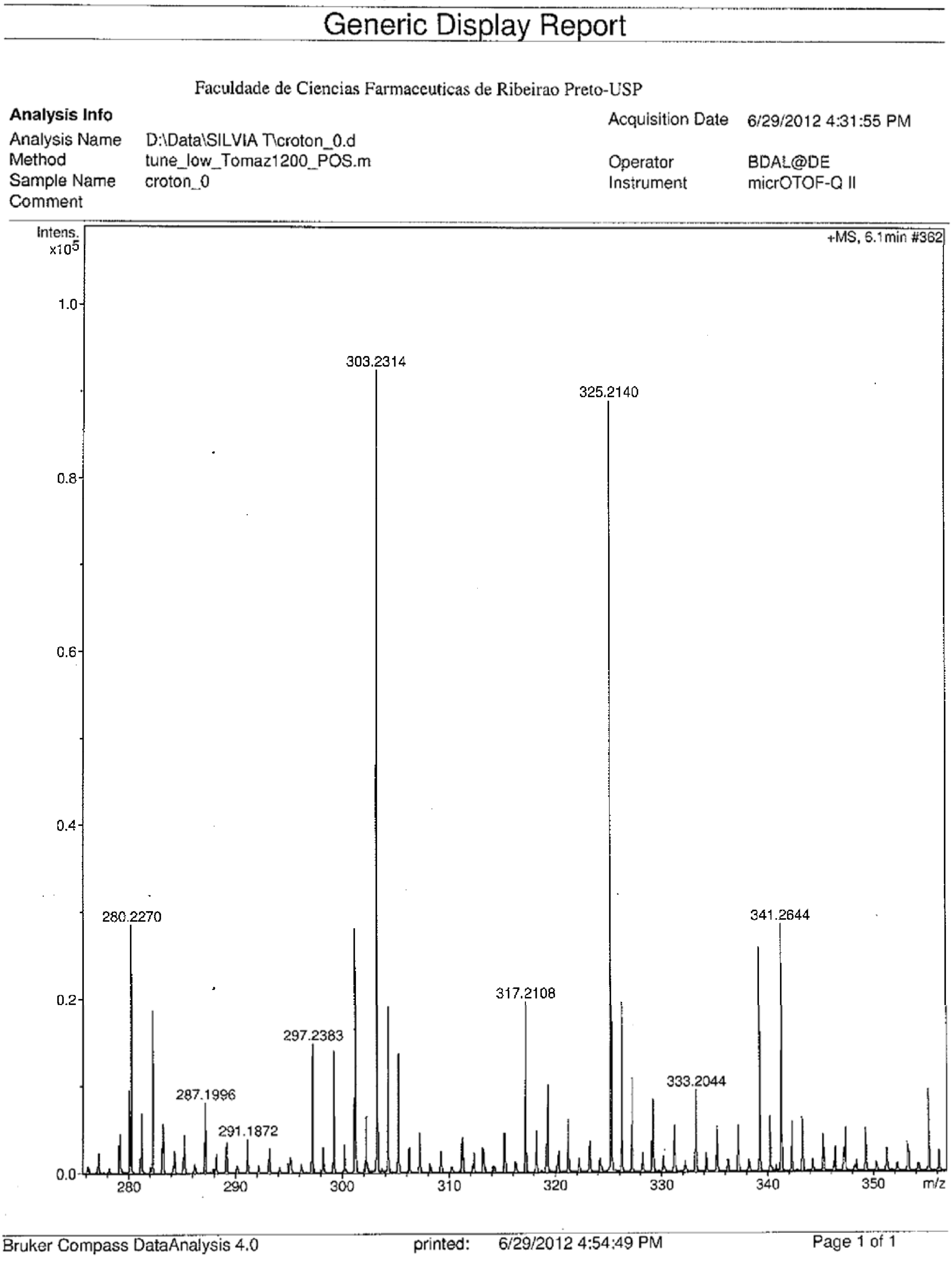

Figure 4. ^{1}H-NMR spectrum of compound **1**.

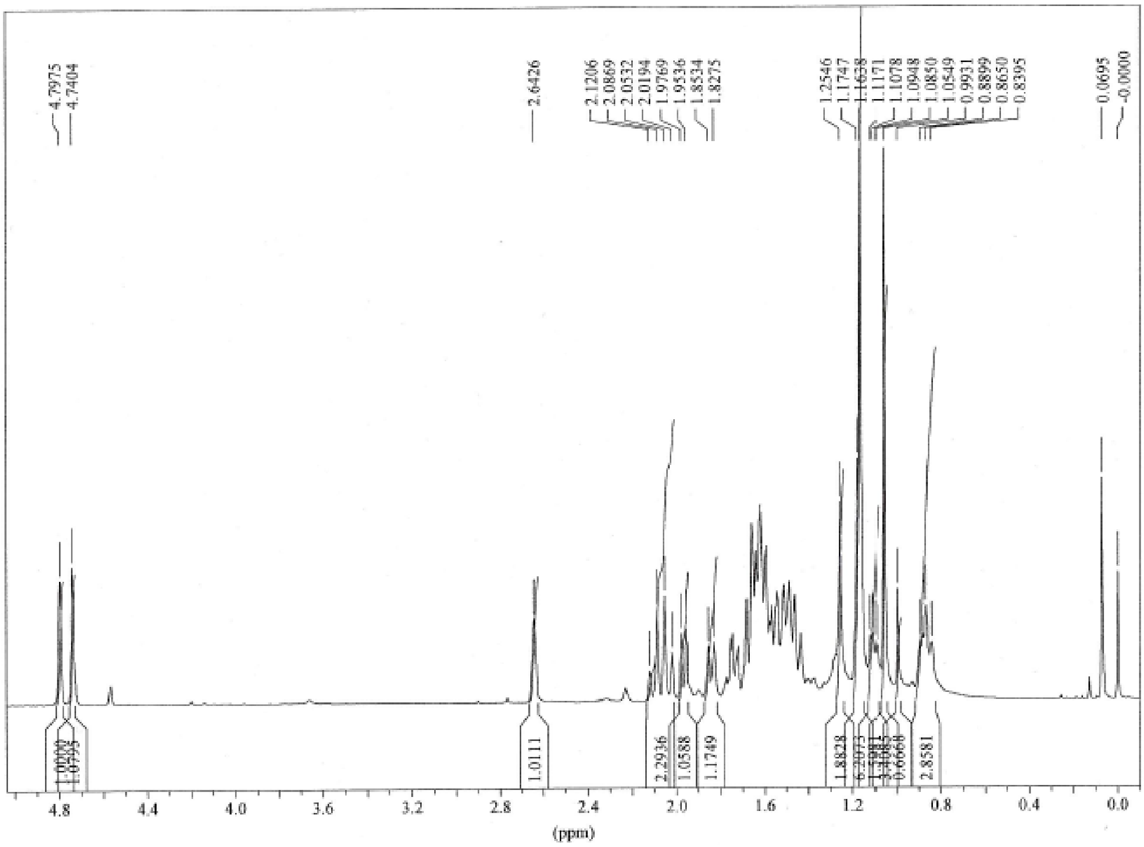

Figure 5. Expansion of ^{1}H-NMR spectrum of compound **1**.

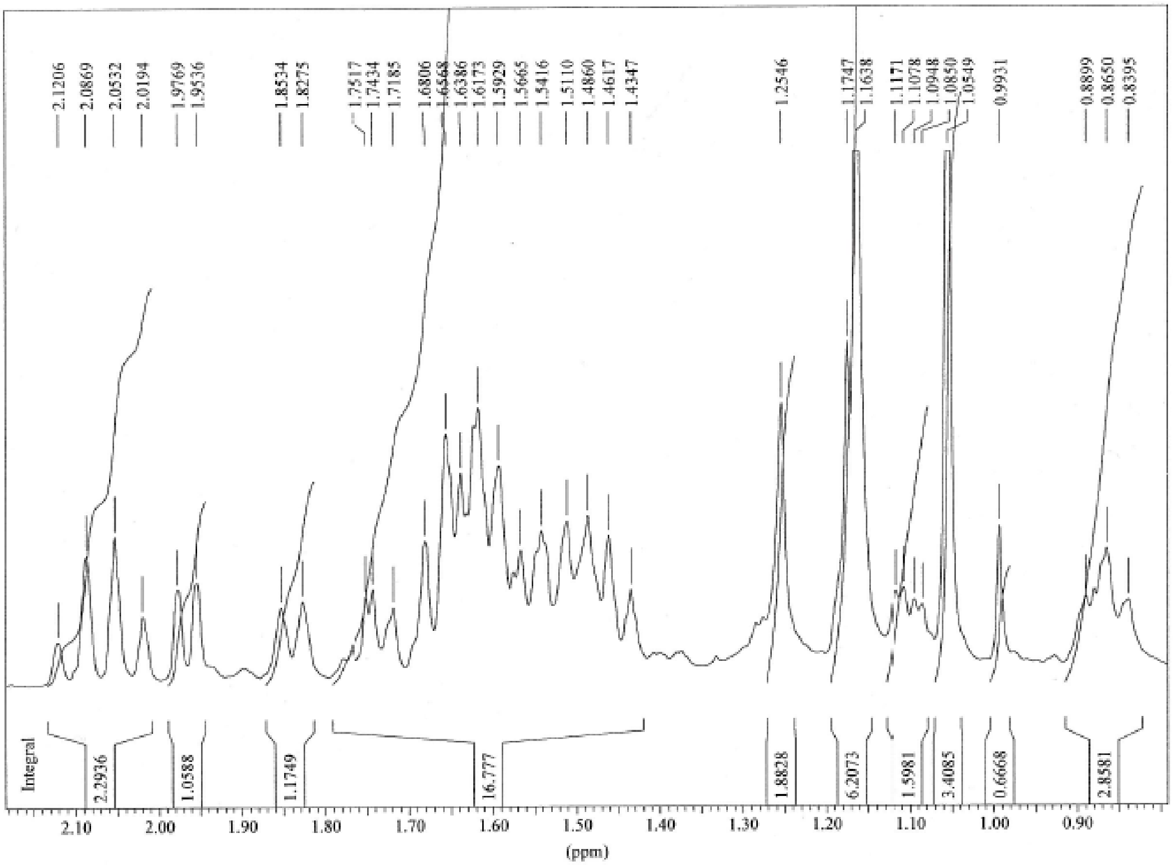

Figure 6. ^{13}C-NMR spectrum of compound **1**.

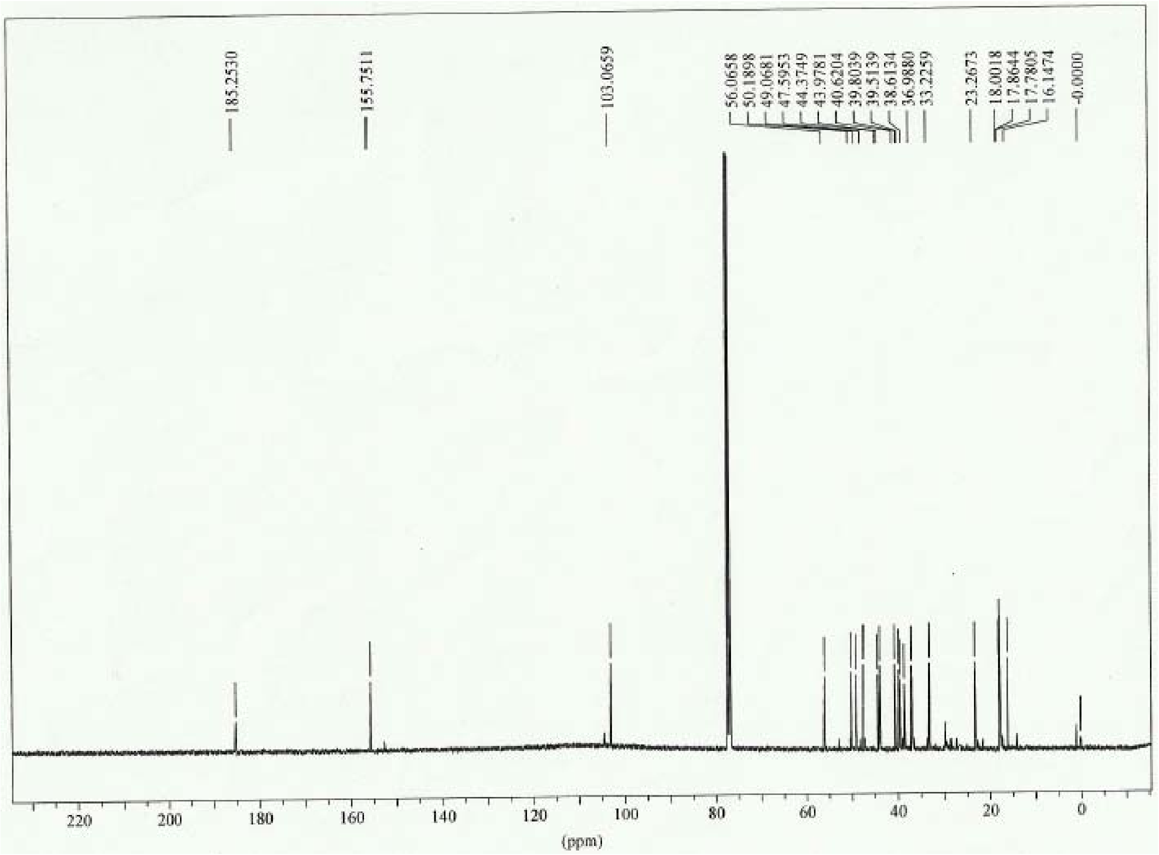

Figure 7. Expansion of ^{13}C-NMR spectrum of compound **1**.

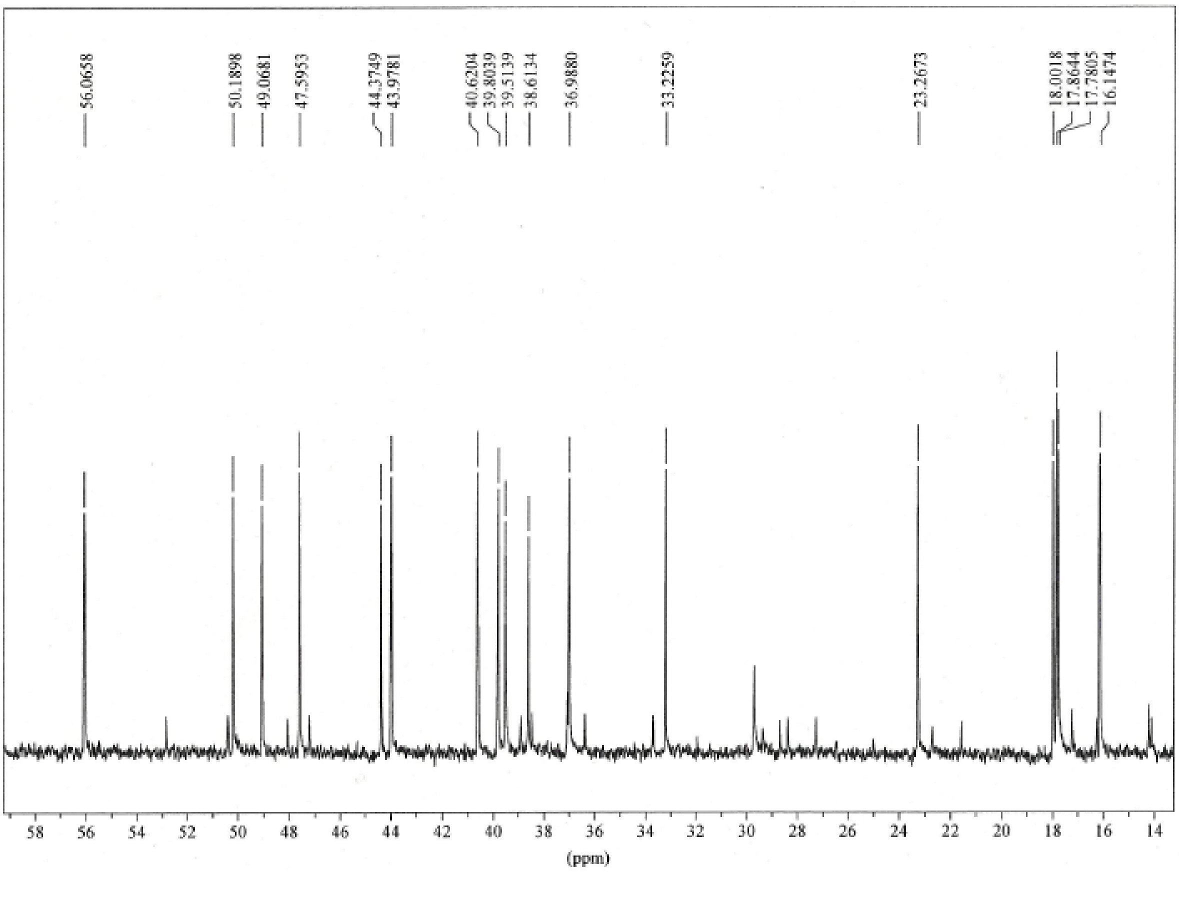

Figure 8. DEPT-135 spectrum of compound **1**.

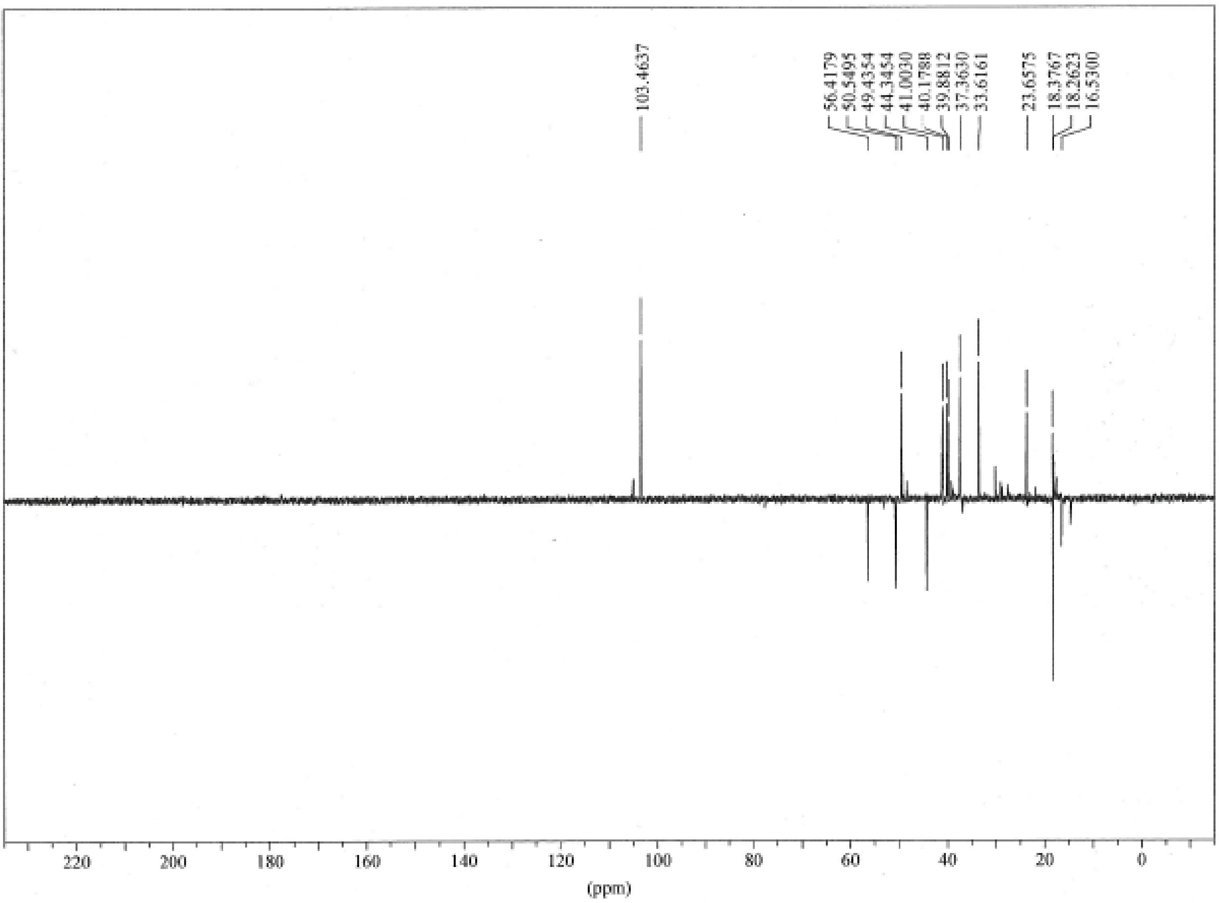

Figure 9. 2D HMQC spectrum of compound **1**.

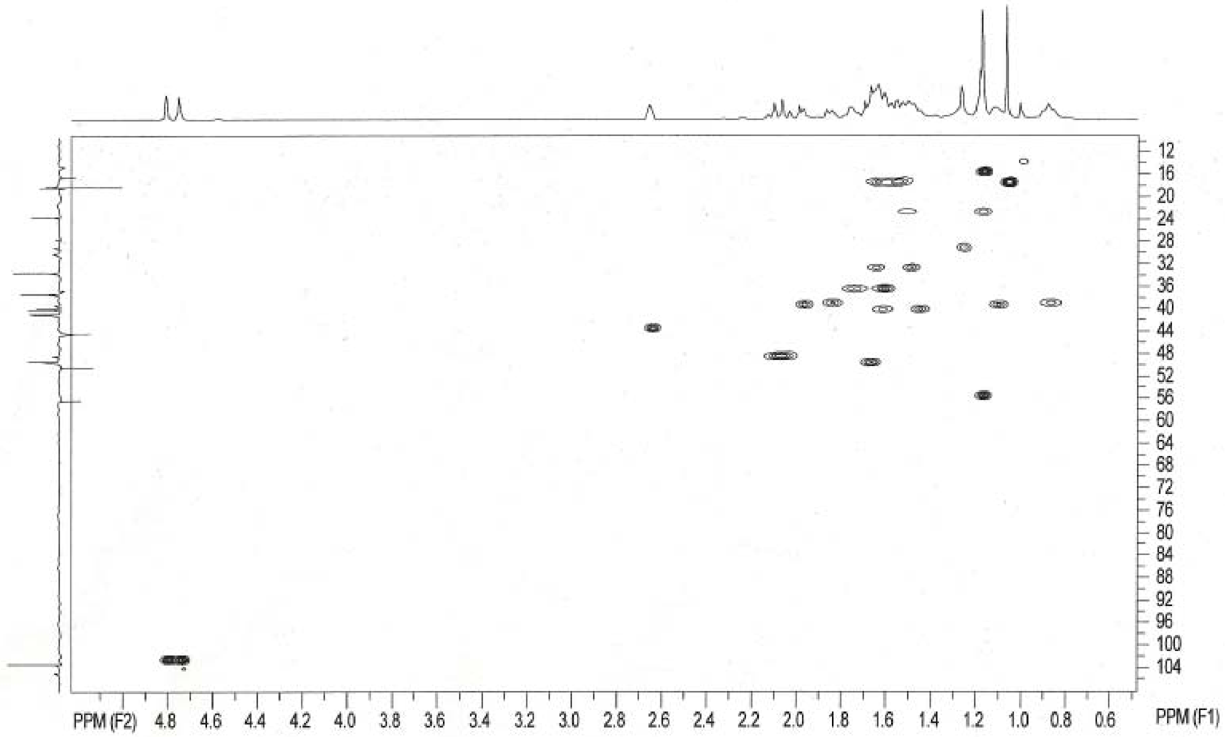

Figure 10. Expansion of 2D HMQC spectrum of compound **1**.

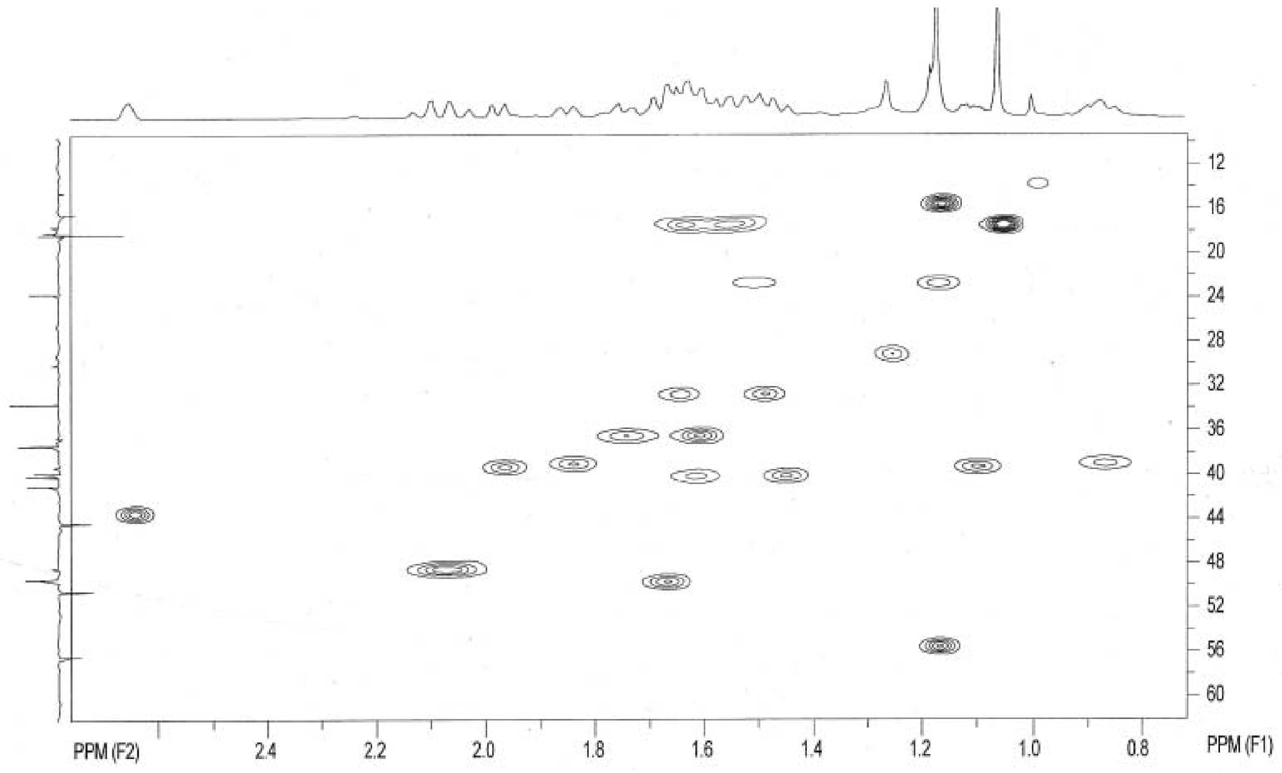

Figure 11. 2D HMBC spectrum of compound **1**.

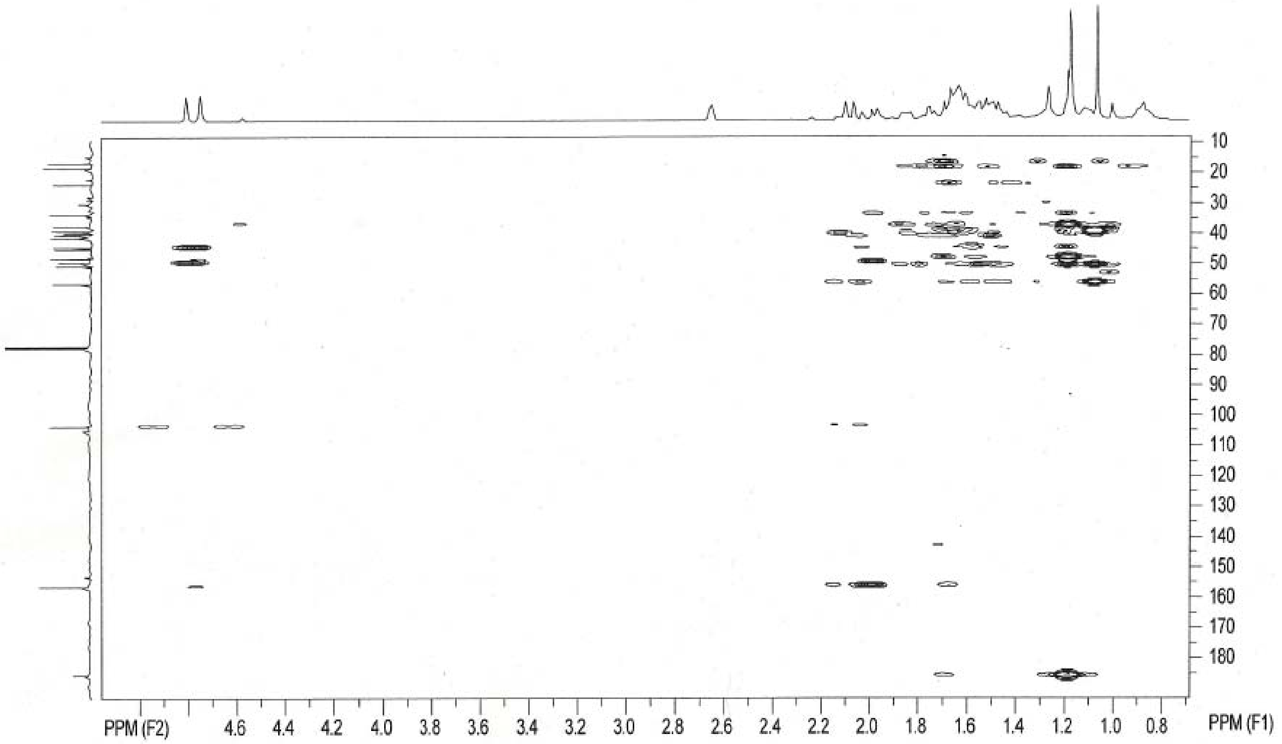

Figure 12. Expansion of 2D HMBC spectrum of compound **1**.

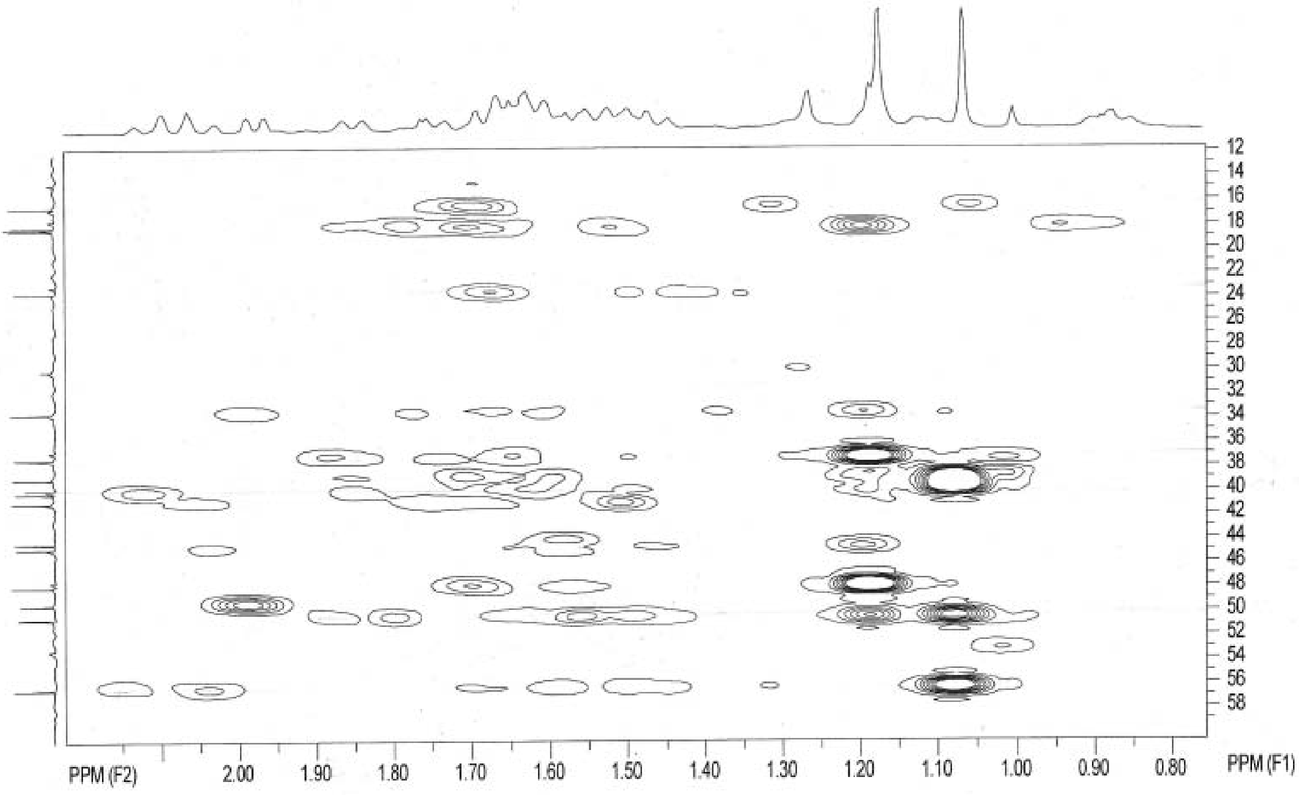

Figure 13. NOEDIFF spectrum of compound **1**.

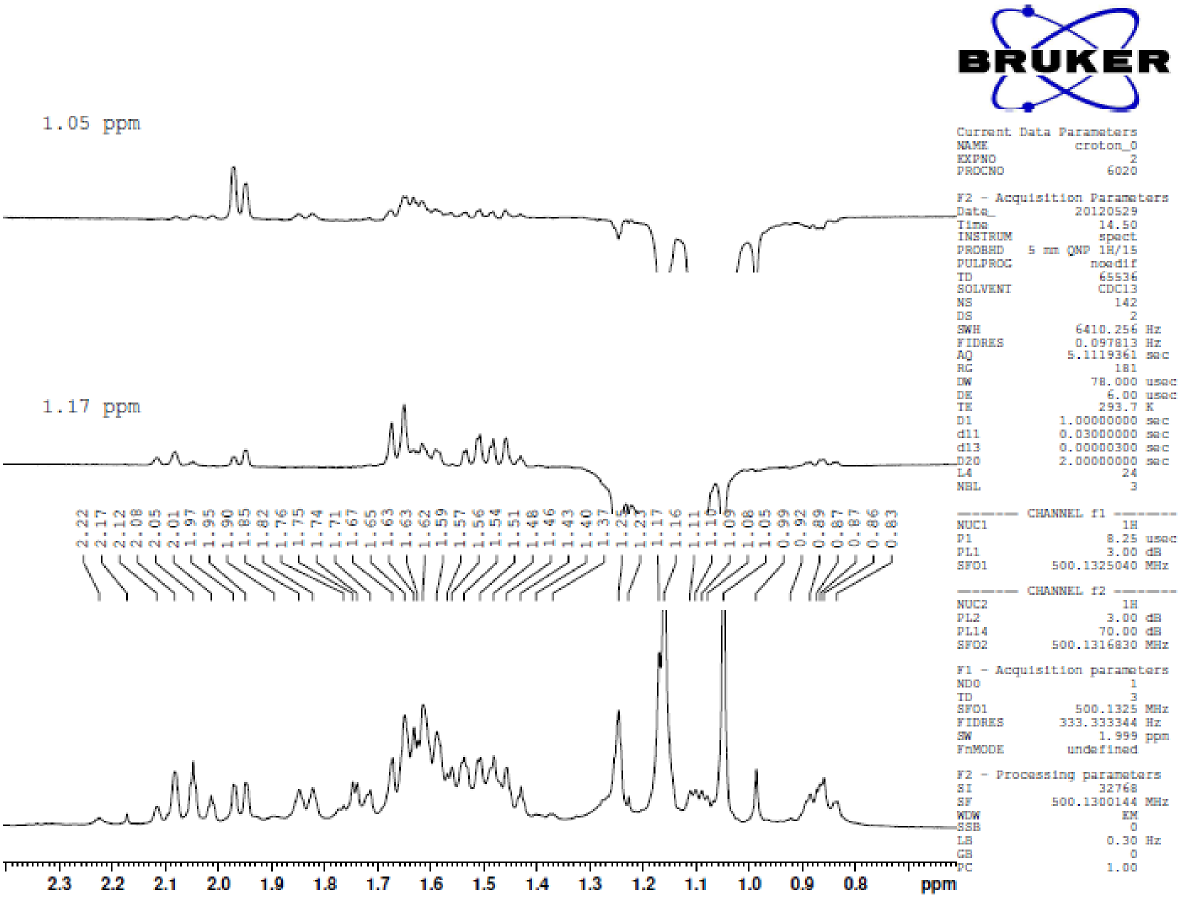

Figure 14. Expansion of NOESY spectrum of compound **1**.

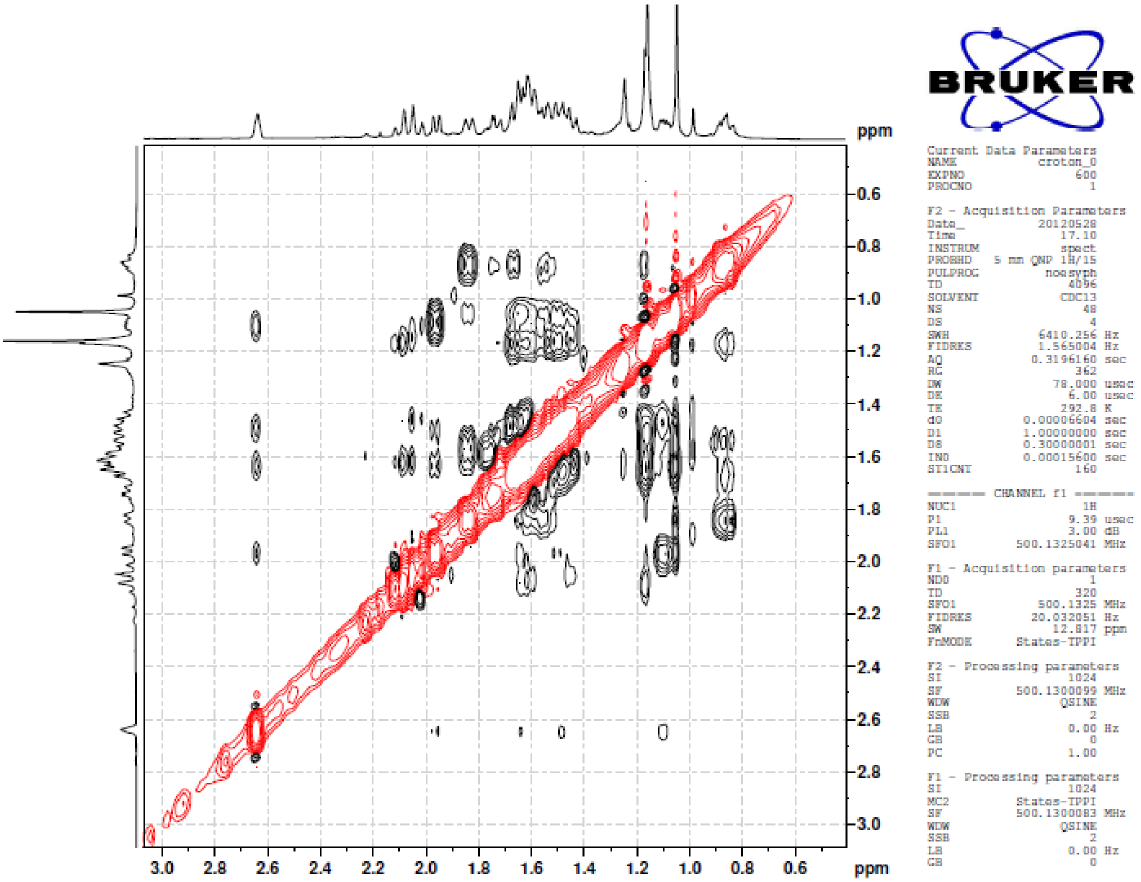

Figure 15. Expansion of NOESY spectrum of compound **1**.

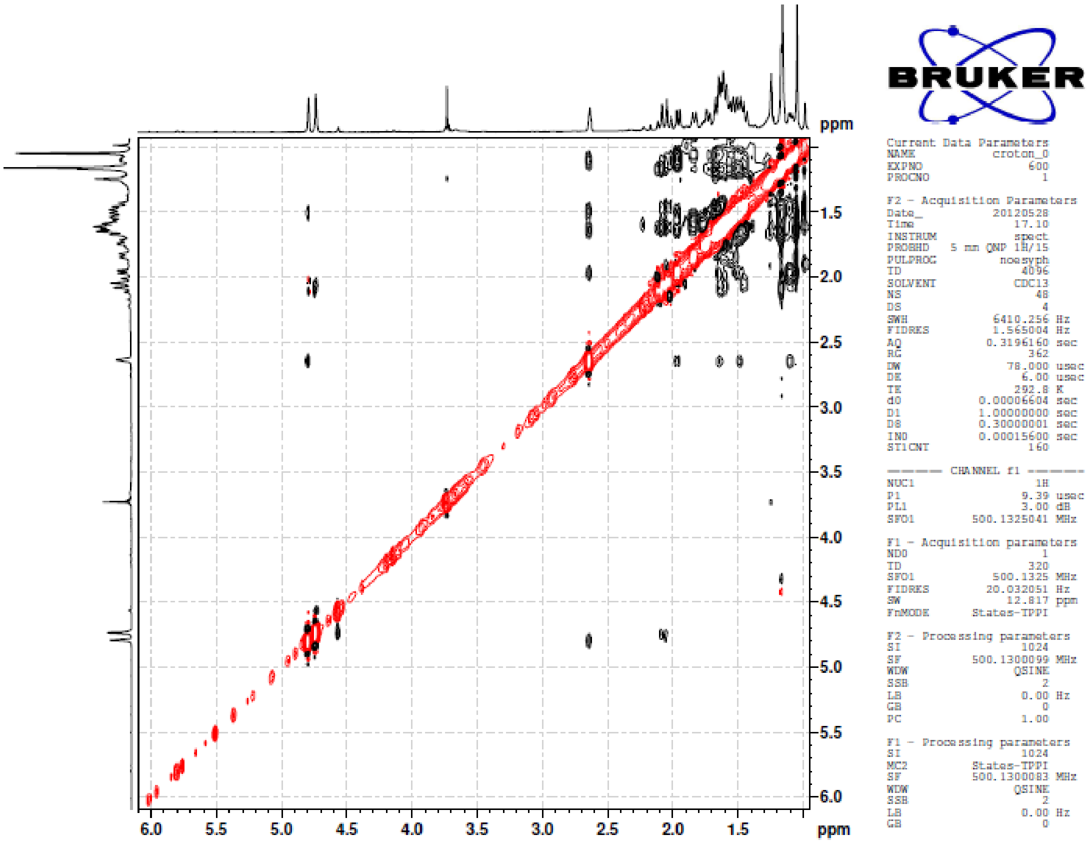

Figure 16. NOESY spectrum of compound **1**.

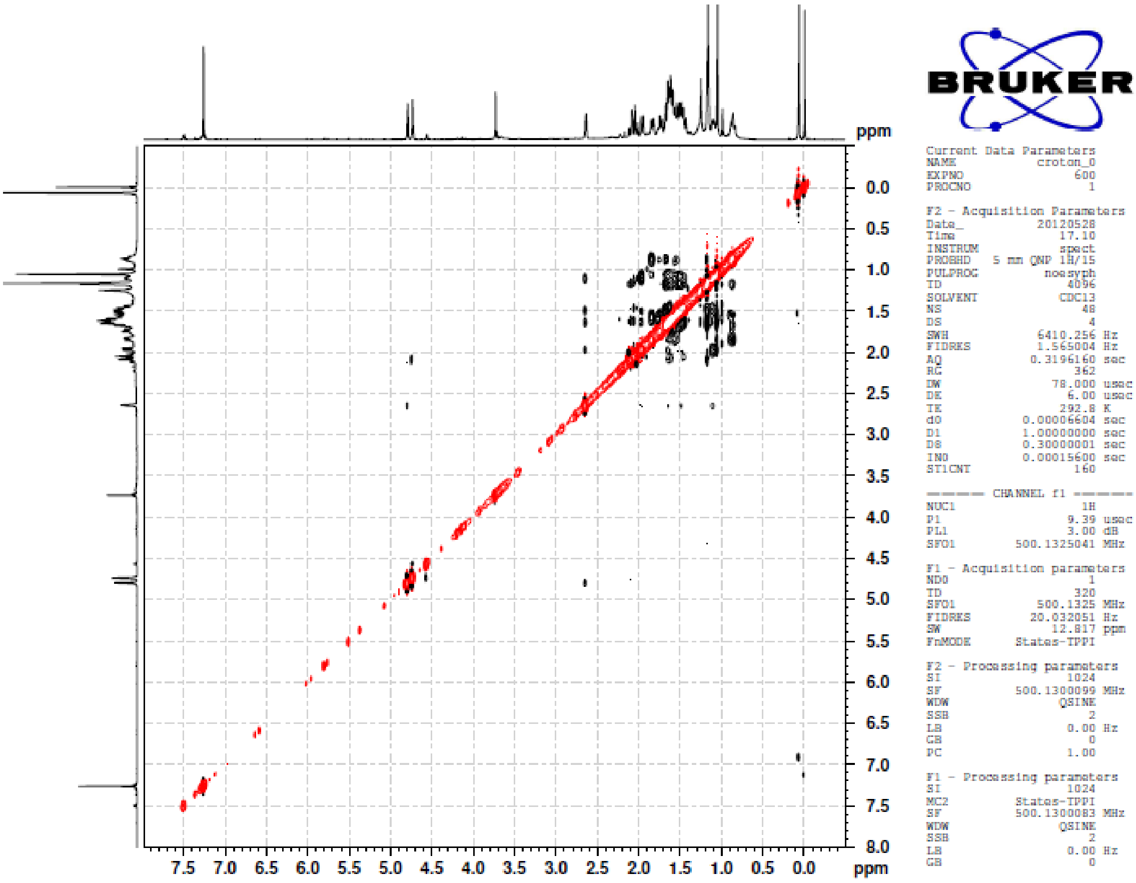

Figure 17. Rows of two-dimensional NOESY spectra of compound **1**.

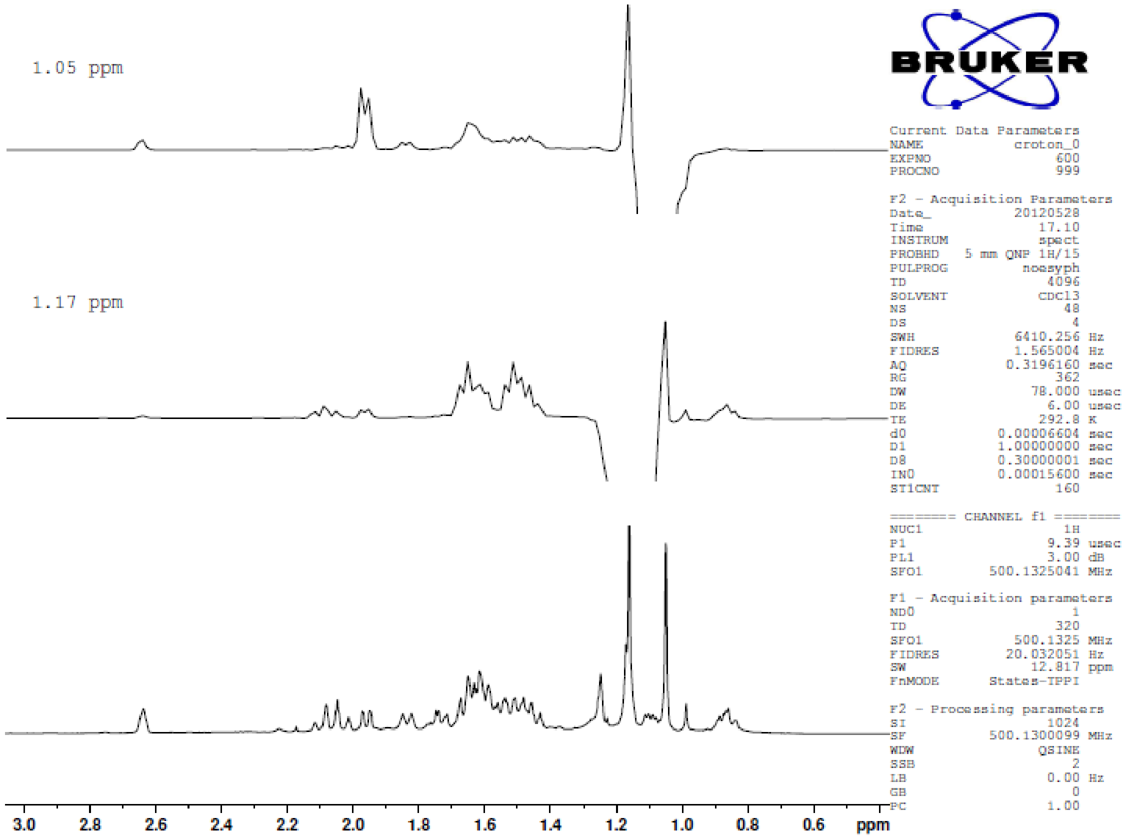

Supplement: Supplementary file 1 [file molecules-17-08851-s001.pdf]
